# Gene Association with Leprosy: A Review of Published Data

**DOI:** 10.3389/fimmu.2015.00658

**Published:** 2016-01-12

**Authors:** Priscila Saamara Mazini, Hugo Vicentin Alves, Pâmela Guimarães Reis, Ana Paula Lopes, Ana Maria Sell, Manuel Santos-Rosa, Jeane Eliete Laguila Visentainer, Paulo Rodrigues-Santos

**Affiliations:** ^1^Faculty of Medicine, Immunology Institute, University of Coimbra, Coimbra, Portugal; ^2^Immunogenetics Laboratory, Department of Basic Health Sciences, Maringá State University, Maringá, Paraná, Brazil; ^3^Immunology and Oncology Laboratory, Center for Neurosciences and Cell Biology, University of Coimbra, Coimbra, Portugal

**Keywords:** leprosy, innate immunity, immune response genes, *Mycobacterium leprae*

## Abstract

Leprosy is a chronic infectious disease caused by an obligate intracellular bacterium known as *Mycobacterium leprae*. Exposure to the bacillus is necessary, but this alone does not mean an individual will develop clinical symptoms of the disease. In recent years, several genes have been associated with leprosy and the innate immune response pathways converge on the main hypothesis that genes are involved in the susceptibility for the disease in two distinct steps: for leprosy *per se* and in the development of the different clinical forms. These genes participate in the sensing, main metabolic pathway of immune response activation and, subsequently, on the evolution of the disease into its clinical forms. The aim of this review is to highlight the role of innate immune response in the context of leprosy, stressing their participation in the signaling and targeting processes in response to bacillus infection and on the evolution to the clinical forms of the disease.

## Introduction

The clinical manifestations of leprosy depend on the interaction between the *Mycobacterium leprae* (*M. leprae*) and the individual’s immune system (1, 2). The indeterminate form of the disease usually appears at the beginning and could evolve into a cure or to any of the clinical forms mentioned below. Depending on the immune profile of the host against infection, the disease may present within a spectrum of clinical manifestations that vary from a localized form [tuberculoid–tuberculoid (TT)] to a disseminated form [lepromatous leprosy (LL)] or one of three intermediate forms (borderline–tuberculoid; borderline–borderline; borderline–lepromatous) (3).

Although the phenotype of susceptibility to infection by *M. leprae* is complex and influenced by factors of the host and the parasite, and also by environmental conditions, some studies have suggested the human genetic factors as important on the acquisition of leprosy and the clinical course of disease (4, 5).

## Pathogen vs. Host

**Figure 1:**
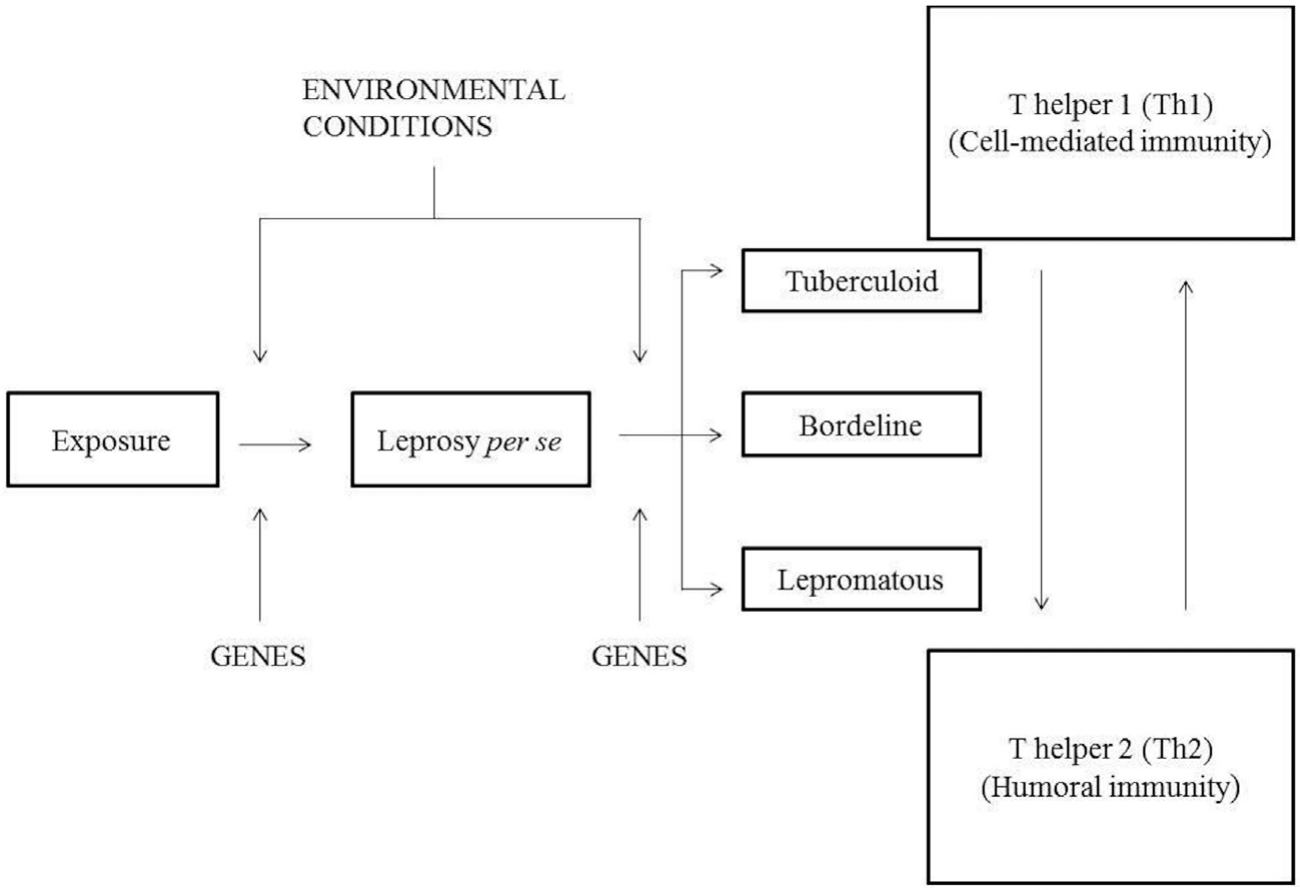
**Model of the leprosy spectrum according to Prevedello and Mira** (8).

Although considerable attention has been focused on the development of the adaptive cellular immune response during the course of infection, recent investigations of the mechanisms and modulation of innate immunity support the idea that after the indeterminate leprosy, immunoregulatory events should occur, which determine the spectrum of disease. The innate immune response, which is the first line of defense against *M. leprae*, is considered a crucial factor in the development of a response against the bacillus as it has essential effector components to combat the pathogen and is able to direct adaptive immunity (6). Most individuals exposed to *M. leprae* do not develop the disease, which may be explained, at least in part, by innate resistance provided by the individual’s genetic background (1), as demonstrated by recent clinical and epidemiological evidence (7). This demonstrates the need of studies investigating genetic markers associated with the disease. The spectrum of leprosy can be represented in the following model (Figure 1) (8).

An individual when exposed to *M. leprae* can develop leprosy *per se* under the influence of environmental and genetic factors that act on the immune response genes determining efficiency in response to infection. If a person develops the disease, it can be taken spontaneously, with good immune response induced by genetic factors or develop the spectrum of leprosy developing any clinical signs: TT, BT, BB, BL, and LL. In the paucibacillary (PB) polo (tuberculoid form, TT), activation of Th1 response occurs from TCD4^+^-activating macrophages to release inflammatory cytokines and proinflammatory that lead to cell-mediated immunity. While multibacillary (MB) pole is driven by Th2 response, which produces anti-inflammatory cytokines, thereby inhibiting macrophage microbicidal function of extending the disease to a pole with high bacterial load. When an individual is exposed to *M. leprae*, he can develop leprosy *per se* by the influence of environmental and genetic factors that act on genes from the immune system, which determines the response efficiency to the infection. If a person develops the disease, it can be taken spontaneously, with good immune response induced by genetic factors, or develop the spectrum of leprosy developing clinical signs: TT, BT, BB, BL, and LL. In the PB polo (tuberculoid form, TT), activation of Th1 response occurs from TCD4^+^-activating macrophages releasing inflammatory and proinflammatory cytokines that lead to cell-mediated immunity. When MB pole is driven by Th2 response, the production of anti-inflammatory cytokines will inhibit macrophage microbicidal function, extending the disease to a pole with high bacterial load.

## Response Innate Immune in Leprosy

As shown in Figure 1, cell-mediated immunity, phagocytes such as macrophages, for example, are activated and have a relevant role in innate immunity. In these cells, as well as neutrophils, natural killer (NK) cells, and some lymphocytes, are present receptors/sensors responsible for the activation of innate immunity, such as *TLRs*, *VDR*, *NRAMP1*, *MRC1*, *NLRs*, *MBL*, *PARK2/PACRG*, *MIC*, *KIR*, cytokines, and *CD14*. Macrophages are phagocytic cells that internalize microorganisms so that they are processed and presented to T cells (TCR) by the MHC molecules, as well as generate inflammatory responses reactions with the release of oxygen and nitrogen species (oxidative bust) and cytokines that can activate other cells of the immune system. T cells, in turn, release IFN-γ that may activate other immune system cells, which are capable of eliminating infected cells. Still, the *NRAMP1* gene is encoded inside the macrophages, lysosome membrane proteins that assist in the process of phagocytosis, acting on ion transport.

**Figure 2:**
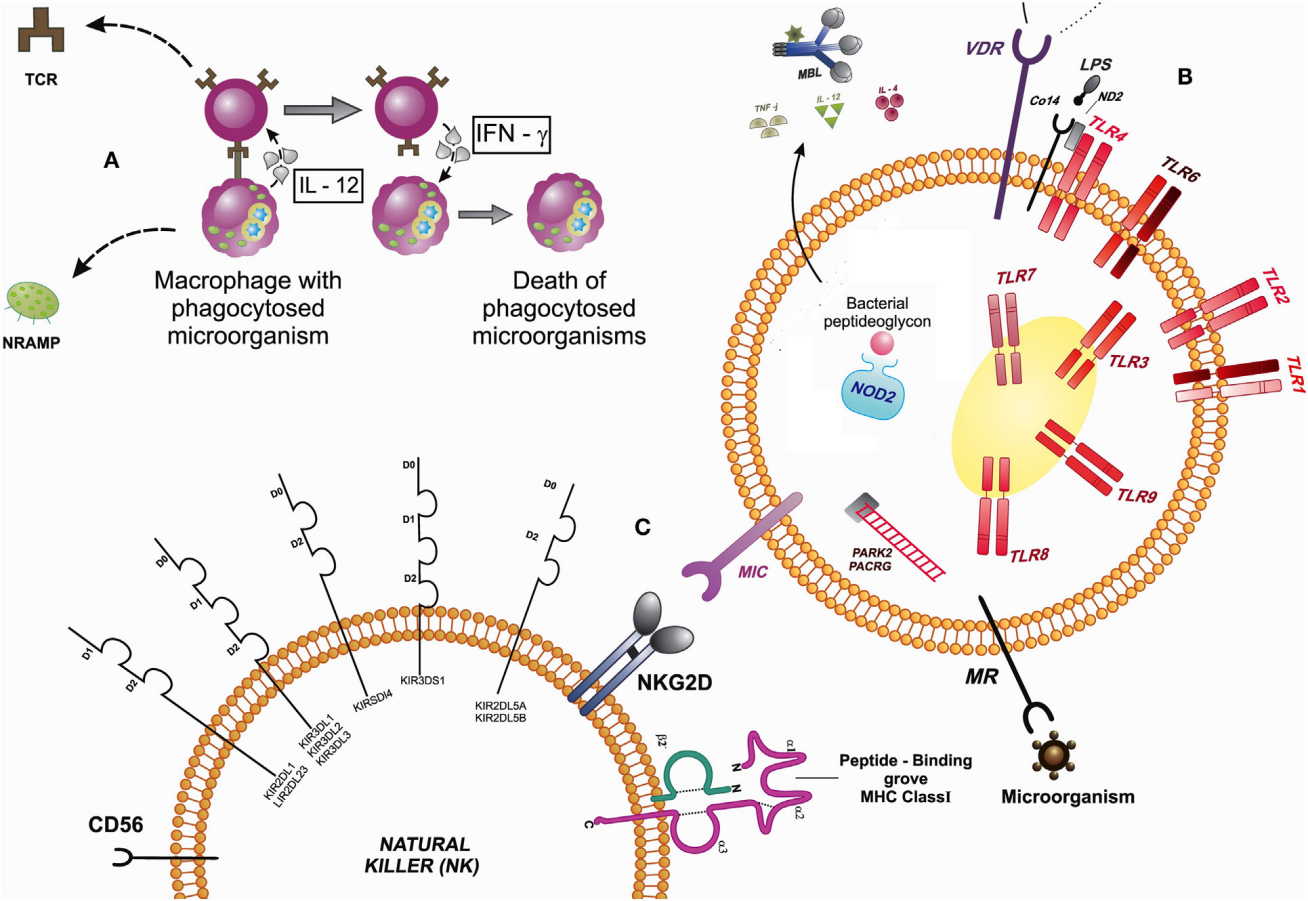
**Signaling between cells that participate in the innate immune response by expressing receptors that interact in the presence of a microorganism**. **(A)** Macrophages are phagocytic cells that internalize microorganisms and presented to T cells (TCR). T cells, in turn, release IFN-γ that may activate other immune system cells. The NRAMP1 gene is encoded inside the macrophages, lysosomal membrane proteins that assist in the process of phagocytosis, acting on ion transport. **(B)** The immune cells have cell surface receptors that can promote a downstream signaling: TLRs, VDR, MRC, and NOD-2 (modulate the autophagy). MIC, which are proteins that are induced into a state of cellular stress, can be recognized by the NKG2D receptor on the surface of Tγδ lymphocytes, CD8^+^ αβ T lymphocytes, and natural killer cells (NK) that contribute to defend the body against infections. The PARK2 acts in the coding process of E3 ubiquitinase necessary signaling cascade in certain cellular processes, such as NOD2, requiring E3 ubiquitin in NF-κB activation process. **(C)** NK cells recognize MIC expressed in infected cells by NKG2D receptor. Abbreviations: TLR, toll-like receptors; MRC, mannose receptor of the C-type lectin; NOD2, nucleotide-binding oligomerization domain 2 protein, human; MBL, mannose-binding lectin; PACRG/PARK2, PACRG protein, human/PARK2-coregulated protein, human; MIC, major histocompatibility complex class I chain-related genes; KIR, killer cell immunoglobulin-like receptors; IL, interleukins; TNF, tumor necrosis factor; IFN, interferons; MHC, major histocompatibility complex; NKG2D, killer cell lectin-like receptor subfamily K; protein; NRAMP, natural resistance-associated macrophage protein; TCR, T-cell receptor; MR, mannose receptor; VDR, vitamin D receptor; LPS, lipopolysaccharide; MD2, myeloid differentiation protein-2, human.

The immune cells have cell surface receptors that can promote a downstream signaling. Among the surface receptors of innate response, there are TLRs, which sense microorganisms and cell activation; VDR, which together with the TLR2 participates in the activation of the vitamin D-mediated antimicrobial pathway, where the vitamin D receptor (VDR) induces the production of antimicrobial peptides, such as cathelicidin; MRC with the TLR and NOD-2 modulate the autophagy and are involved in host–pathogen interactions; They are important in the sensing of mycobacterial peptides; MIC, which are proteins that are induced into a state of cellular stress, and can be recognized by the NKG2D receptor on the surface of Tγδ lymphocytes, CD8^+^ αβ T lymphocytes, and NK cells that contribute to defend the body against infections. Other TLRs receptors are expressed in endosome compartments, and there are still receptors present in the cytoplasm as NOD2 and RIG1, acting as cytosolic sensors in the presence of bacteria and viruses, which might have escaped the intracellular internalization. These receptors recognize peptides while releasing cytokines and activate the complement inflammatory system and therefore cytokines for adequate inflammatory response. The PARK2 acts in the coding process of E3 ubiquitinase necessary signaling cascade in certain cellular processes, such as NOD2, requiring E3 ubiquitin in NF-kB activation process. NK cells recognize MIC expressed in infected cells by NKG2D receptor. These cells also express killer cell immunoglobulin-like receptor (KIR) molecules for activation and inhibition, as well as MHC class I and II, that can recognize peptides and to activate T lymphocyte. Markers characteristic of these cells (NK) are CD56 (Figure 2).

### Toll-Like Receptors

**Figure 3:**
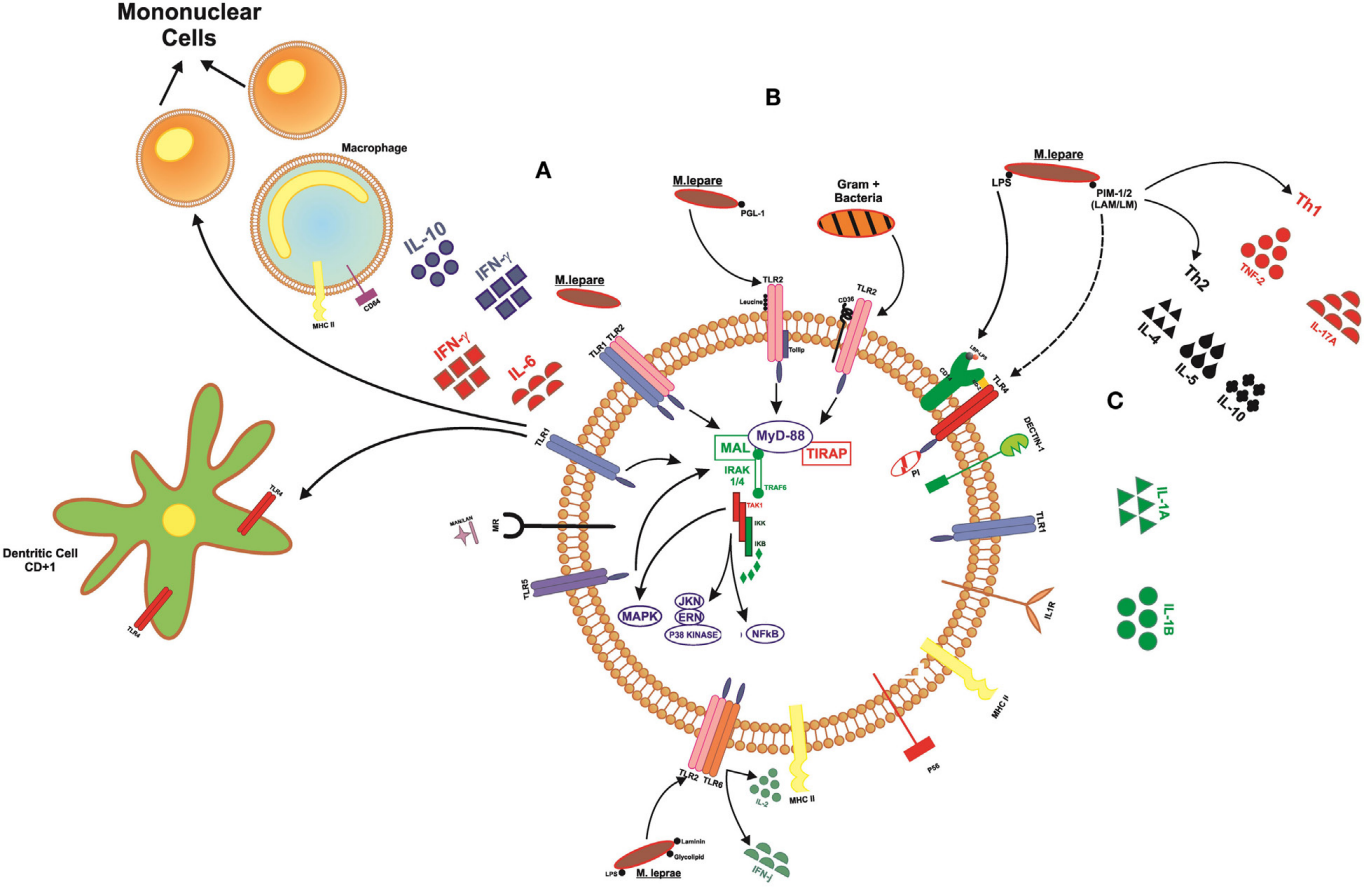
**Signaling toll-like receptors and interaction with cells of the immune system**. **(A)** TLR1 acts on the differentiation of mononuclear cells into macrophages, releasing cytokines, such as IFN-γ and IL-6, whereas when activated, macrophages can release IFN-γ and IL-12. This receiver also operates in the maturation of dendritic cells, which become able to present the peptide to lymphocytes T. **(B)** TLR2 heterodimer with other markers, such as TLR1, TLR6, CD36, Dectin1, being able to recognize membrane components of bacteria Gram^+^ and Gram^−^. **(C)** TLR4 forms a complex with MD2 in response to LPS. The marker CD14 binds to LPS. This LBP–LPS complex binds and releases the TLR–MD2 complex to initiate intracellular signaling.

Pattern recognition receptors (PRRs), stand out in the innate immune response, such as Toll-like receptors are present on the cell surface (TLR1, TLR2, TLR4, TLR5, and TLR6), sensing microbial components such with lipids, lipoproteins, and proteins and also in the cytoplasm (TLR3, TLR7, TLR8, and TLR9) sensing microbial and viral species of nucleic acids. The receptors TLR1/2 are the first to act by promoting cell activation in the presence of *M. leprae* (9, 10). TLR1 recognize lipoproteins present in bacillus and promote the differentiation of monocytes in macrophages with the release of cytokines, such as IFN-γ and IL-6, and also affect the maturation of dendritic cells CD1b^+^, which can activate other effector cells of the immune system, such as T cells, promoting an anti-microbicide activity (11–14) (Figure 3).

Some *TLR* genes indicate influence of the development of mycobacterial diseases because they are able to detect different molecules in the bacillus *Mycobacterium* sp. such as lipomannan, lipoarabinomannan, phosphatidylinositol mannoside, laminin, LPS, and lipoprotein glycolipids. Once in contact with the TLR receptor, a signaling cascade is initiated within the host cell. Therefore, alterations in these genes may confer susceptibility/resistance to leprosy development.

*TLR1* N248S polymorphism was studied in a population of Bangladesh. The homozygous genotype SS was associated with leprosy, but as a protective factor to the development of ENL-type reactions, and the heterozygous genotype SN was associated with protection against leprosy (15). In populations of Brazil, case–control and family studies have been performed with 3,162 individuals showing an association between *TLR1* 248S and leprosy; corroborating the finding that 248S is a susceptibility factor for leprosy (16). A case–control study also investigated the *TLR1*-I602S SNP in three distinct populations: New Delhi, Kolkata, and Turkey; the authors reached the same conclusions thereby demonstrating consistency in the data obtained (17). SNPs in other genes of the *TLR* family have also been investigated and their mechanisms of action have been evaluated. One study reported the analysis of the *TLR1* gene (N602S) and the *TLR6* gene (G1083C), both of which were associated with increased IFN-γ levels. The *TLR6* (G1083C, C745T) was associated with increased IL-2 levels, whereas *TLR1* (A1188T) was associated with elevated IFN-γ and IL-2 levels (18). Three polymorphisms in *TLR2* (597C > T, 1350T > C, and a microsatellite marker) were analyzed in 431 Ethiopian patients with leprosy and 187 control subjects. The microsatellite and the 597C > T polymorphisms both influenced susceptibility to reversal reaction (19). The influence of GT microsatellite on the expression of TLR2 was measured in 88 leprosy patients, 95 household contacts, and 96 healthy controls. The allele/genotype of TLR2 microsatellite that includes longer GT repeats was associated with low TLR2 mRNA expression and high IL-10 production, while that the shorter GT repeats were associated with high TLR2 mRNA expression and low IL-10 production. High IL-10 producing allele of TLR2 microsatellite might predispose household contacts to leprosy (20). One study on the *TLR4* gene (896GA, 1196CT) in an African population showed a protective effect of 896GA and 1196TT against the development of the leprosy (21). Additionally, this study suggested that polymorphisms in the *TLR2* gene increase the risk of a patient developing reversal reactions; however, this polymorphism was not considered a risk factor for the development of leprosy *per se*. These reports on different ethnic groups reinforce the idea that *Toll-like* gene receptors participate in the outcome of leprosy.

### Vitamin D Receptor

*TLR2* participates in the activation of the vitamin D-mediated antimicrobial pathway, where the VDR induces the production of antimicrobial peptides, such as cathelicidin. Polymorphisms in the *VDR* gene can destabilize and/or modify mRNA activity of the VDR, resulting in susceptibility to intracellular pathogens (22). This vitamin–receptor interaction leads to the suppression of several genes, such as interleukin-12 (*IL12*), colony-stimulating factor 2 (*CSF2*) coding granulocyte-macrophage colony-stimulating factor (GM-CSF), interferon-gamma (*IFNG*), and *HLA-DRB1*, inhibiting the production of cytokines and immunoglobulins, and lymphocyte proliferation (22–24).

Various polymorphisms in the *VDR* gene are being investigated for interfering in the mRNA activity and transcription. Polymorphisms located near the 3′UTR of the *VDR* gene (*Bsm*I, *Apa*I, and *Taq*I) are related to the stability/transcriptional activity of *VDR* mRNA, while a polymorphism located in the translation initiation codon, *Fok*I, gives rise to a three amino acid difference in the VDR length that affects protein function (25). *Taq*I polymorphism in the *VDR* gene can occur by a substitution of a C nucleotide for T, with the loss of a *Taq*I restriction site in the gene, resulting in “T” (wild) and “t” (normal) alleles. A study of the population of Mexico associated the *TT* genotype in the *VDR* gene to the lepromatous form of the disease (26), and also in the population of Calcutta, the *tt* genotype was associated with Tuberculoid form leprosy, and the *TT* genotype was associated with Lepromatous form. Heterozygotes with the *Tt* genotype were found associated with protection against both leprosy types (23). Although a study of the *Taq*I polymorphism of the *VDR* gene in the population of Minas Gerais, Brazil, did not find significant differences, there was a higher frequency of the “t” allele in MB compared to PB patients and control individuals, suggesting the participation of this allele in developing the most severe form of the disease. In this study, a positive association was also observed between the *tt* genotype and a negative Mitsuda test in patients with leprosy (22). In the population of Malawi, homozygotes with the *tt* genotype with a silent T > C change in codon 352 of the *VDR* gene are susceptible to the disease (27). These different results can be the consequence of the diverse allele and genotype frequencies among populations.

### Natural Resistance-Associated Macrophage Protein 1 Gene

The *NRAMP1* gene, located in the chromosome 2q35 region, has been reported as one factor responsible for the resistance of mice to intracellular pathogens (28). In human cells, it is expressed in macrophages and encodes a protein found in lysosomal membranes that, in the process of phagocytosis, is recruited for phagosome membranes containing pathogens, where it acts as a transporter of iron and other divalent ions. Iron is essential for biological functions, both for host immune defense and mycobacterial growth.

In a study performed in an endemic region of Brazil, there were no significant differences in the allelic and genotypic frequencies of the *NRAMP1* gene in relation to the Mitsuda test among patients and household contacts, nor between those with the MB and PB forms of the disease. However, individuals with a negative lepromin response associated with genotype “22” or “23” presented sevenfold and eightfold greater chances of developing leprosy, respectively (29). A sib-pair linkage analysis between the Mitsuda response and the *NRAMP1* gene was done among 20 nuclear families with leprosy from Ho Chi Minh City, Vietnam. All family subjects were genotyped for several intragenic and flanking *NRAMP1* markers, leading to the definition of a fully informative *NRAMP1* haplotype. Significant linkage was observed between *NRAMP1* and Mitsuda reaction when considered either as a quantitative (*P* < 0.002) or as a categorical (*P* = 0.001) trait. Separate analyses among healthy and affected sibs showed evidence for linkage in both subsamples, indicating that linkage between the Mitsuda reaction and *NRAMP1* is independent of leprosy status (30).

Hatta et al. determined the association of three polymorphic variants (*D543N*, *3*′*UTR*, and *INT4*) of the *NRAMP1* gene with tuberculosis and leprosy in 58 tuberculosis patients, 42 leprosy patients and 198 healthy controls from South Sulawesi, Indonesia. An association of the *INT4* polymorphism was observed with the PB type of leprosy (31). In a study conducted in Mali, West Africa, a total of 273 patients with leprosy and 201 controls were genotyped for *NRAMP1* polymorphisms previously associated with tuberculosis. No association was found with leprosy *per se*, but the *NRAMP13*′*UTR* polymorphism was associated with leprosy subtypes (32).

The *INT4*, *D543N*, and *3*′*UTR* polymorphisms of *NRAMP1* were also analyzed by in patients with leprosy from Thailand. There were no significant difference in the distribution of the genotypes and allele frequencies of *NRAMP1* polymorphisms between the patients and controls (33). The data from seven multi-case leprosy families (84 individuals) from French Polynesia were analyzed by Roger et al. Nine polymorphic loci and three microsatellite markers (*D2S104*, *D2S173*, and *D2S1471*) within *NRAMP1* gene were typed and the results observed suggest that *NRAMP1* is not linked to leprosy susceptibility in the French Polynesian families tested (34). Another study analyzed polymorphisms of this gene to investigate susceptibility for leprosy reactions in Recife, Brazil (*274C/T*, *D543N*, and *1729* + *55del4* polymorphisms of the *NRAMP1* gene). The mutant *274TT* genotype prevailed in cases without reversal and ENL reactions, suggesting the 274 C/T polymorphism of the *NRAMP1* gene in determining the susceptibility to reactions in individuals with leprosy (35). Members of 20 multiplex leprosy families of Vietnam and China (*N* = 168) were genotyped for *NRAMP1* alleles and 4 closely linked polymorphic markers and the finding showed that the segregation of *NRAMP1* haplotypes into affected siblings was significantly non-random (36).

### Mannose Receptor C-Type 1 Gene

The *MRC1* gene, located in the chromosome 10p13 region, encodes the mannose receptor (MR) in humans. TLRs, the nucleotide-binding oligomerization domain (*NOD*)-2 and *MRC1* genes modulate the autophagy and are involved in host–pathogen interactions; they are important in the recognition of mycobacterial peptides and activation of signaling intracellular. A study of Vietnamese families analyzed the G396S polymorphism in exon 7 of the *MRC1* gene and suggested that it is a protective factor against leprosy *per se* and MB leprosy (37). In a Brazilian population, the *G396-F407* haplotype was considered a risk factor, while the *S396-F407* haplotype was considered a protective factor against leprosy (37). Subsequently, Wang et al. analyzed the polymorphism of genetic variants of the *MRC1* and *IFNG* genes in Han Chinese patients with leprosy. Although the results have not been confirmed, the rs692527 and rs34856358 variants of the *MRC1* gene were found to be associated with PB leprosy, and the rs3138557 variant of the *IFNG* gene was associated with MB leprosy (38).

### Cytosolic Receptors: Nucleotide-Binding Oligomerization Domain Protein (NOD1 and NOD2)

Several studies have correlated autophagy with protecting host against various intracellular bacteria that use different strategies to establish infection. After phagocytosis of the infectious agent, different molecules are encoded, such as Toll-like receptors, the cytosolic Nod-like receptors (NLRs), and RIG-I-like receptors (RLRs), all of which are important in the detection of intracellular pathogens. In innate response, the cytosolic sensors belonging to the NLR family are able to perceive disturbances in the membrane caused by microorganisms and directly recognize MDP mycobacterial components. Some NRLs activate NF-kB pathway through inflammasome (ASC/Caspase-1), releasing IL-1 and IL-18, while others NRLs, such as NOD2 activates NF-kB recruiting RIPK2, TRAF, and E3-Ubiquitin, triggering a signaling cascade to activate IFN regulatory factor (IRF) and IKKs, and following of P38, JNK, ERK MAPK activation, releasing IFNα/β and other proinflammatory cytokines. NOD1 is able to direct immune response to Gram-negative bacteria, while NOD2 recognizes Gram-negative and Gram-positive (14). Among inflammatory cytokines, NOD2 induce Th17 responses that, in turn, keep the inflammatory response. Th17 cells produce IL-17A, IL-17F, IL-21, and IL-22 as their signature cytokines. IL-17 is correlated with induction of proinflammatory programs associated with chemokine secretion and neutrophil recruitment that cooperate for mycobacterial killing (39). Inflammatory cytokines, such as TNF-α, IL-12, and IL-4, are produced after the recognition of the microorganism and other components as nitric oxide. Thus, the NOD2 plays an important role in the response to microorganisms, helping in the recognition, autophagy, and modulating the immune response.

Several studies have correlated *NOD2* gene polymorphisms to leprosy based on its role in the recognition of mycobacteria shortly after signaling mediated by TLRs. Two SNPs in the *NOD2* gene were found to be associated with leprosy in a study of 706 Han Chinese patients and 1226 healthy controls: rs9302752 (OR = 2.28; IC = 1.70–3.06) and rs7194886 (OR = 2.25; IC = 1.58–3.21) suggesting risk for development of leprosy (40). In India, the rs9302752 SNP was investigated in 211 leprosy patients and 230 controls, but the results were not considered because they were not in Hardy–Weinberg equilibrium (41). The study of Zhang (2009) (40) was replicated in a Chinese population with tuberculosis (1043 patients vs. 808 controls) for the SNPs: rs3135499, rs7194886, rs8057341, and rs9302752 (42). No significant differences were found between the genotype frequencies of patients and controls, although when they analyzed patients with bacteriological confirmation test, the variant rs7194886 showed risk to disease (CT/TT vs. CC: OR = 1.35; IC = 1.05–1.72) (42). For the NOD2 SNPs found to be associated with leprosy among Chinese patients (Zhang, 2009) (40), only the rs9302752 SNP was replicated in 474 samples family based from Vietnamese with PB and MB leprosy (*N* = 188 and *N* = 286, respectively) suggesting risk for development of severe form of leprosy (MB) (*P* = 0.014; OR = 1.27; IC = 1.27–1.58) (43). Marques et al. (44) describe the results of a validation and replication GWAS study of the Chinese (Zhang, 2009) (40) in Brazilians, using a stepwise strategy that involved two family-based and three independent case–control samples, resulting in 3,614 individuals. Three *NOD2* alleles of rs8057341 (A allele, Pc = 0.003), rs2111234 (G allele, Pc = 0.031), and rs3135499 (C allele, Pc = 0.023) were statistically significant in family-based study association, indicating protection to leprosy (44). The replication of the association study between *NOD2* rs8057341 in different populations from Brazil (Rondonópolis, Bauru, and Rio de Janeiro) was observed in all case–control samples, with the AA genotype conferring resistance to leprosy (*P* = 1.39 × 10^−6^; OR = 0.49; IC = 0.36–0.65), but the same results were not observed in the family-based study. To obtain an overall estimate, all samples (case–control and family-based studies) were included to build a summary plot that indicated a consensus protective OR value (A allele = 0.80, *P* = 0.0001), confirming allele A of *NOD2* rs8057341 as a leprosy resistance genetic factor (44). Another study in 933 patients (240 patients had reversal reactions and 124 had ENL reactions) of Nepal compared with 101 controls, suggested that NOD2 genetic variants are associated with susceptibility to leprosy and the development of leprosy reactive states. Four polymorphisms were identified as significant (*P* < 0.05) at the allelic level: (rs12448797: OR 2.18; rs2287195: OR 1.51; rs8044354: OR 1.53; and rs1477176: OR 0.44). Genotypic analysis revealed that these associations with ENL were significant for seven SNPs suggesting susceptibility to developing of type II reactions: (rs2287195, rs8044354, rs7194886, rs6500328, rs17312836, rs1861759, and rs1861758) (45). Singh et al. examined the occurrence of the three most common polymorphisms in the *NOD2* gene (Arg702Trp, Gly908Arg, and 3020insC) in patients with mycobacterial diseases, but significant differences were not observed (46). However, a study performed in mononuclear cells of individuals homozygous for the 3020insC NOD2 mutation, showed an 80% defective cytokine response after stimulation with *M. tuberculosis*. It was also demonstrated that TLR2 and NOD2 mycobacterial ligands synergize for the production of inflammatory cytokines, and that this synergism is lost in cells lacking either of these receptors. The intracellular pathways induced by NOD2 and TLR2 during recognition of mycobacterial components synergize, and the stimulation of cytokine production by *M. tuberculosis* is greatly impaired in individuals with NOD2 mutations (47). Analysis of the sequence variation in the coding regions of the *NOD2* gene in the 377 African-American from Houston, TX, USA, patients with tuberculosis and the 187 control subjects identified 56 sequence variants. Transition substitutions were more prevalent (74.5%) than transversions (25.5%) among these SNPs. Three SNPs (Pro268Ser, Arg702Trp, and Ala725Gly) demonstrated significant associations with tuberculosis. Minor allele carriers (heterozygous and homozygous) of Pro268Ser (OR = 0.55; CI = 0.32–0.94; *P* = 0.02), and Arg702Trp (OR = 0.27; CI = 0.08–0.88; *P* = 0.01) presented decreased risks for tuberculosis. Conversely, the minor allele carrier (heterozygous) of Ala725Gly showed an increased risk for tuberculosis disease (OR = 2.16; CI = 1.10–4.72; *P* = 0.03) (48).

### Mannose-Binding Lectin

Mannose-binding lectin (MBL) is a soluble protein in the serum, which participates in the innate immune response with activation of the complement system and opsonization effects. This protein binds to several pathogens, allowing complement-mediated lysis and linkage to membrane of mycobacteria. Many studies found an increase in the concentrations of MBL in leprosy patients (25, 49, 50).

The genetic analysis of a study developed with a population of Nepal (933 leprosy patients), which examined polymorphisms in the *TNF* and *MBL* genes showed the *MBL-G161A* variant associated with protection against LL, and identified a polymorphism associated with low MBL levels (homozygosity of *MBL*-G161A), which was associated with a reduced risk of LL when compared with TT leprosy (OR = 0.33; CI95% = 0.12–0.85, *P* = 0.010) (25). In Southern Brazil, the polymorphisms at the promoter and exon 1 regions of the *MBL2* gene were assessed on 264 patients with leprosy and 214 matched healthy control subjects and the results showed a decreased frequency of haplotypes/genotypes associated with low expression of circulating MBL in lepromatous patients when compared with tuberculoid patients. The *LYPA* haplotype related with high expression of MBL was associated with susceptibility to leprosy *per se* and to progression to the lepromatous and borderline forms of the disease (51). However, another study in the Brazilian population (228 patients) investigated SNPs of the *MBL2* gene and did not find significant differences in genotype and allele frequencies (49). Finally, a recent study, in the Chinese population, analyzed the ficolin-2 (FCN2), mannose-binding lectin (MBL2) and complement factor H (CFH) genetic variants, which may influence the innate immune response *to M. leprae* (50).

### PARK2/PACRG

Parkinson’s disease is characterized by the participation of the *PARK2* gene and its coregulator *PACRG* both with a length of 2 Mb in the chromosome 6q 25.2-q27 region. These genes share a regulatory overlap region of about 5 kb, as well as a common bidirectional promoter (52). The *PARK2* and *PACRG* genes encode proteins that are involved in cellular metabolism of ubiquitination. *PARK2* encodes parkin and an ubiquitin E3 ligase that is involved in the release of polyubiquitinated proteins to the proteasome complex. It is suggested that ubiquitin-mediated proteolysis has an important role in the control of infections by *M. leprae*.

Four SNPs in the promoter region of the *PARK2* gene and adjacent to the *PACRG* gene were found to be associated with leprosy in a study of Vietnamese families. Furthermore, in the same study using a high-density association scan of both genes, 19 SNPs between intron 1 of the *PARK2* gene and intron 2 of the *PACRG* gene showed evidence of association. The study was replicated in the Brazilian population and the findings were confirmed (53). Recently, findings reported by Alter et al. confirm that the SNPs identified in the promoter region of *PARK2*/*PACRG* are associated with leprosy in two independent and ethnically distinct samples, thus supporting the evidence that these variants are risk factors for the development of the disease (54). This study evaluated a Vietnamese (198 single cases) and an Indian (364 cases) population and a control group of northern India (370). In the Vietnamese sample, 69 SNPs were associated with leprosy, and as expected, two SNPs (rs1040079 and rs9356058) confirmed the study carried out by Mira et al. (53). In the case–control study in India, three SNPs were also found associated in the Vietnamese population (rs6915128, rs10806768, and rs1333955) (54). However, analysis of 11 previously indicated risk variants and other 2 SNPs (rs13195186 and rs1801474), which are all located in the region of *PARK2* and *PACRG*, shows no significant association with susceptibility to leprosy *per se* in the Chinese Population (55). In an Indian population, Malhotra et al. investigated the association of six SNPs present in this regulatory region in 286 leprosy patients and 350 healthy controls and no association with leprosy susceptibility was observed (56).

Recently, conducted a mapping of *PARK2* and regulatory region of the *PACRG* gene in the north Indian and east Indian-Orissa population groups, where 11 of the 96 SNPs studied showed a strong association with susceptibility to leprosy. The results showed 10 SNPs in the region of *PARK2*: rs9347683 within the core promoter region of *PARK2* gene and SNPs rs9347684, rs9346929, rs4709648, rs12215676, rs10806765, rs6936373, rs1333957, rs9365492, and rs9355403, located within 63.8 kb upstream region of the *PARK2* gene and only one SNP (rs10945859), located 6.67 kb upstream of *PACRG* gene (57).

### Major Histocompatibility Complex Class I Chain-Related Genes

Major histocompatibility complex class I chain-related genes A (*MICA*) and B (*MICB*) are located on chromosome 6 near to the HLA-B and HLA-C loci. These genes are highly polymorphic producing different MICA and MICB proteins which are induced by cellular stress. MICA molecules are recognized by the NKG2D receptor on the surface of γδ T lymphocytes, CD8^+^αβ T lymphocytes, and NK cells that contribute to defend the organism against infections, including against infection by *M. leprae* (58, 59).

To determine the association of *MICA* gene with leprosy and its subgroups, Wang et al. examined 69 Southern Chinese patients and 112 healthy controls. The frequency of the MICA*A5 allele showed a decreasing tendency in the patients with leprosy as compared to the controls, but the difference was not significant. However, the frequency of the MICA*A5 allele was significantly decreased in the MB patients but not in the PB patients (60). A study analyzed exon 5 of the *MICA* gene and intron 1 of *MICB* in families of Southern India who developed PB leprosy and showed that MICA*5A 5.1, MICB*CA16 and MICB*CA19 were associated with the disease (58). The MICA*5A5.1 allele, associated with leprosy susceptibility in this much larger study, has a single G insertion occurring in a background of five alanine repeats causing a frame shift mutation that results in a premature stop codon and a truncated transmembrane domain (61).

Recently, our group conducted a study of *MICA* genes and leprosy in a population of southern Brazil showing that the MICA*027 allele was decreased in leprosy *per se* and in MB patients (62). MICA*027 codifies a transmembrane domain, A5, related to the normal expression of protein associated with protection against, suggesting susceptibility toward leprosy and its most severe form.

### Killer Cell Immunoglobulin-Like Receptor Genes

Natural killer cells, which participate in innate and adaptive immunity, can be cytotoxic when activated by an exaggerated expression of ligands for the activation receptors on the surface of target cells. The KIRs expressed on the surface of NK cells interact with other cells through specific HLA molecule ligands, transmitting activating or inhibitory signals (63). These receptors possess HLA molecules as ligands and can act by activating the cell, through activating receptors, or inhibit the cell through inhibitory KIR. The main HLA ligands are divided into three groups: C1, C2, and Bw4. The HLA ligands of the C1 Group are characterized by presenting serine at position aa77 (AGC) and asparagine at position aa80 (AAC) and binding to KIR2DS2, KIR2DL2, and KIR2DL3 receptors. The C2 Group is characterized by presenting asparagine at position aa77 (AGC) and lysine at position aa80 (AAA) and binding to the KIR2DS1 and KIR2DL1 receptors. The Bw4 Group acts as a ligand to the KIR3DL1 receptor (64).

A study of *KIR* and their ligands was carried out in a Brazilian population comparing leprosy patients with a control group for the first time by our group (65). The activating receptor genes *KIR2DS2* and *KIR2DS3* were found at higher frequencies in the group of patients with tuberculoid leprosy compared to the lepromatous group. The frequency of *KIR2DL1* with its ligand C2 (HLA-C*02, *04, *05, *06, *07, *15, *17, and *18) was lower in the group with the borderline form of the disease, compared to the control group and to patients with tuberculoid form. Moreover, there was a lower frequency of *KIR2DL3* with C1 (HLA-C*01, *03, *07, *08, *12, *13, *14, and *16) in patients with tuberculoid leprosy compared to control individuals, to patients with leprosy *per se* and to the borderline group (65). Other study realized with population from Rondonópolis, Center-East Brazil, there was a higher frequency of activating *KIR* genes, KIR2DS1, 2DS2, and 3DS1 and your ligands HLA in the tuberculoid (TT) group as compared to the LL group. In this same study, KIR2DL2/2DL2-C1 was more frequent in the patient, TT and LL groups than in the control group. Higher frequency of inhibitory pairs it was present in Borderline patients when compared to the control group, and a higher frequency of activating pairs as compared to the LL group. It was realized multivariate analysis and confirmed the associations and demonstrated that being a female is a protective factor against the development of the disease *per se* and the more severe clinical form (66).

### Cytokine Genes

The substitution of a single base in cytokine genes may cause differences in the expression of these molecules generating structural and functional changes which may influence the host’s response to a pathogen. Several authors also described polymorphisms of cytokine genes associated with the clinical forms of leprosy (67–70).

Brazilian researchers (67, 69–72) found an association between the *TNF*-308A (rs1800629) allele and a protective effect against the development of leprosy and results similar were also observed in a study in Nepal in 2010 (25). A study performed in Rio de Janeiro by Vanderborght et al. (73) observed the relationship between the A allele in the promoter region of the *TNF*-308 and a lower bacteriological index (BI), whereas the A allele in the promoter region of the *TNF*-238 (rs361525) was associated with a higher BI. However, in Mexican population (74), no association was found between *TNF-*308G/A and leprosy. Some studies have suggested a protective role for TNF-α in TT and BT patients (75). A study, conducted in an Indian population, showed that the frequency of the *TNF2* allele (with substitution of G > A at position -308, the *TNF* gene promoter region) was significantly associated with LL. A Thai study also showed an increase in the frequency of the allele in MB patients, indicating that this allele in this position induces susceptibility to the more severe forms of the disease (33). However, Levee et al. (76) found no link between *G1M*, *G2M*, *KM*, *IL1B*, *TNFA* (1, 2), and *TNFA* (A, G) and leprosy when performed a linkage study with six families of French Polynesian. In the multi-case leprosy family study from northeastern of Brazil, the common allele TNFA1 of the -308 promoter region polymorphism showed linkage and/or association with disease *per se*, at a high level of significance. Two loci transmission disequilibrium testing suggested susceptibility to TNFA1/LTA2 and protective to TNFA2/LTA2 haplotypes in the class III region, suggesting together the segregation and HLA analyses suggest the possibility of more than one susceptibility locus to leprosy in the MHC (68).

A study of an Indian population reported the participation of variants *BAT1-LTA-TNF-BTNL2*, as risk factors for the development of leprosy (77). According to authors, the combination of low T-cell inhibition status of *BTNL2*, less inhibition of TNF-α by *BAT1*, and low TNF-α expression may provide protection from leprosy, which may be stronger in the presence of high TNF-α producer allele genetic background (77). SNPs located at *BAT1* (HLA-B-associated transcript 1) promoter and 13 kb upstream to *LTA* gene affected the transcription factor binding site; hence, the gene expression. BAT1 protein is also known to down regulate pro-inflammatory cytokines, such as TNF-α, IL-1, and IL-6 (78).

IFN-γ, a cytokine secreted by Th1 CD4^+^, CD8^+^ T cells, and NK cells, acts in the body’s defense against intracellular pathogens. A study in a Brazilian population showed that the T allele at position + 874 of the *IFNG* gene conferred a protective effect to leprosy (79), but, Wang et al. (38) found no association between *IFNG* + 874T/A and leprosy in Chinese patients. Nevertheless, the variant rs3138557 in the *IFNG* gene had many CA repeat alleles and they observed that the alleles *IFNG* (10CA), *IFNG* (13CA), and *IFNG* (15CA) had a higher frequency in patients, especially in MB, and that the allele *IFNG* (17CA) was more frequent in PB patients. In Brazilian patients from Amazonas state, there were no significant differences between patients and control group. Though, the A/A genotype and the allele *IFNG* (16CA) were significantly associated with PB compared to MB patients (80). In another population of the same country, Reynard et al. (81) observed that a higher frequency of alleles *IFNG* (15CA), *IFNG* (16CA), and *IFNG* (17CA) was associated with development of leprosy, which indicates that the *IFNG* gene polymorphism may contribute to the course of infection.

Interleukin-6 (IL-6) is a pleiotropic cytokine that plays an important role in a wide range of processes, such as immune response, acute phase reactions, and hematopoiesis (82). In a case–control study (83) was observed a correlation between plasma levels of IL-6 and *IL6* genotypes in patients with Type-2 reactions in leprosy. Thus, the identification of genetic factors predictive of leprosy reactions could contribute on prevention strategies.

Interleukin-12 consists of two covalently linked subunits: p35 and p40. The effects of IL-12 are mainly controlled by the level of transcription of p40 and expression of IL-12R. IL-12 is produced quickly after infection and acts as a proinflammatory cytokine by inducing IFN-γ production and enhancing the proliferation and cytotoxicity of NK and T cells (84). In Indian patients (85), subjects with leprosy were less likely to have the 3′UTR genotype associated with lower IL-12B expression. In a study in Western Mexico (86), it was found that the 1188A/C polymorphism in the 3′UTR of IL12p40 gene was associated with greater susceptibility to LL, without regard to the expression levels of IL-12 p40. On the other hand, Jesús Salvador et al. (87) in a study with Mexican patients found no significant association between genotype and allele frequencies of the 1188A/C polymorphism and LL (87). Liu et al. conducted a multiple-stage genetic association study in leprosy patients from China and observed associations implicating IL18RAP/IL18R1 and IL12B as susceptibility genes for leprosy (88). In another study, Ali et al. observed that SNP rs2853694 (A/C at intron 3) in IL12B gene showed a positive association with leprosy in Indian patients (89).

Interleukin 10 (IL-10) is a cytokine produced by monocytes and activated T cells. It is deeply involved in the regulation of inflammatory and immunological reactions. Several polymorphisms have been observed in the *IL10* gene, including 6–11 CA repeats microsatellite polymorphisms, and three point mutations: -1082 (G/A) (rs1800896), -819 (C/T) (rs1800871), and -592 (C/A) (rs1800872) (90).

In Mexican patients (74), no significant difference was found in the frequency of *IL10*-819C allele in patients and controls. Nevertheless, in a Brazilian population (91), the *IL10*-819T allele was associated with leprosy in both a case–control study and in a meta-analysis. In another Brazilian population (69), where the *IL10*-819TT genotype was significantly higher in patients than in healthy controls and the frequency of the *IL10*-819T SNP was greater in PB patients compared to MB or among control subjects. However, the genotypes C/C and C/T in the SNP -819 and C/C and C/A in the -592 SNP were positively associated with leprosy in a Colombian population. The haplotypes -819C-592C and -1082A-819C-592C showed significant association (92). In another study (93) with Brazilian patients, the haplotype *IL10*-3575A/-2849G/-2763C was associated with resistance to leprosy and development of more severe forms of the disease, and the haplotype *IL10*-3575T/-2849A/2763C with susceptibility to LD. Malhotra et al. (94) in a Indian population observed that the extended haplotype *IL10*-3575T/-2849G/-2763C/-1082A/-819C/-592C conferred resistance to leprosy *per se* and to development of more severe forms of disease, whereas the haplotype *IL10*-3575T/-2849G/-2763C/-1082A/-819T/-592A was associated with the risk of severe form of the disease. A study performed in a population of Southern Brazil, Franceschi et al. (70) showed a lower frequency of haplotype *IL10*-1082G/-819C/-592C in patients with the LL form compared to the control group. These studies suggest the involvement of SNPs in the promoter region of the *IL10* gene in leprosy.

### CD14

Some components of mycobacteria, as PGL-1, are recognized by TLR1/2 heterodimer, while the LPS, present in abundance in the membrane of *M. leprae*, is transferred by CD14 associated with lipopolysaccharide-binding protein (LBP) to the TLR4/MD2 complex. Thereby, it initiates the signaling for intracellular activation of NF-κB, with consequent production of interferon (IFN) and other cytokines, such as IL-6, TNF-α, and IL-1 (9, 10, 14).

CD14 exists in two forms, its soluble form, sCD14, and mCD14, the cell membrane form. sCD14 may be produced by monocytes, hepatocytes and endothelial cells and the functional relevance of sCD14 is its mediation of bacteria-induced cell activation of mCD14 expressing cells (95, 96). In addition to the recognition and internalization of LPS, the differentiation of monocytes to macrophages is accompanied by regulation of CD14 and data suggest that CD14 is essential for TNF-α production, this being the most proinflammatory cytokine produced during bacterial infection. In combination with the TLR4/MD2 complex, CD14 recognize LPS, resulting in a proinflammatory immune response through TIRAP-MyD88-dependent and TRAM-TRIF dependent TLR4 activation pathways (97, 98). The TLR4 is a signaling receptor to LPS and MD2 is a coprotein required to intracellular signaling TLR4 (99).

CD14-159 C/T polymorphism was investigated in several populations with tuberculosis (95, 100–104). A meta-analysis was performed with this marker and -159T allele was associated with risk of development of the disease (OR = 1.27; IC = 1.01–1.61). The genotype TT also suggests risk when compared to genotype CT and CC (OR = 1.52; IC = 1.11–2.08). The results of this meta-analysis suggest that CD14 -159C/T polymorphism is associated with a predisposition to the development of tuberculosis (105).

Pacheco et al. analyzed the -159C/T polymorphism in patients with tuberculosis and a control group of Medellin, Colombia (267 patients vs. 112 controls) but did not find significant results for this polymorphism (95). Another study examined polymorphisms in *NRAMP -INT4*, *MBL* genes (codons 52, 54, and 57) and CD14 -159C/T in patients with tuberculosis in the Caucasian population from Poland, and although it has not obtained statistically significant results, suggested the involvement of CD14 and MBL molecules in the host–mycobacteria interactions on the basis of the significant increase in the serum CD14 and MBL in patients with tuberculosis (100). The same polymorphism (-159C/T) was analyzed in Mexican population, simultaneously with the TLR4 expression on the surface of monocytes. Higher levels of mCD14/sCD14 and TLR4 were observed in tuberculosis patients compared with the control group (*P* < 0.05). The frequency of CD14 -159TT genotype was higher in patients than in control group (35.6 vs. 12.3%). Patients who were homozygous for the T allele had a significantly higher risk for the development of pulmonary tuberculosis (OR = 2.26) (106). In a Korean population (274 tuberculosis patients and 422 healthy controls), the frequency of -159TT genotypes was found higher in tuberculosis patients than in healthy controls. Serum sCD14 levels were higher among tuberculosis patients with -159TT genotypes than among those with -159CC genotypes and IFN-γ release by PBMCs was decreased in subjects with -159TT genotypes (101).

In Chinese population (432 Chinese patients with tuberculosis and 404 controls), two positions were analyzed: CD14 -1145 and CD14 -159. Both the frequency of allele T in the -159 polymorphism (OR = 1.4) and G allele in the -1145 (OR = 1.51) were significantly more frequent in cases than in controls (102). This study confirms the findings in other populations, such as the Americans (107), the Singaporeans (108), and the Chinese (109).

In a case–control study with 698 patients tuberculosis and 404 controls in the Han Chinese population, SNPs in the promoter region do *CD14* gene were analyzed: G1619A, T1359G, A1145G, and C159T, but statistically significant differences were found just for the SNP A1145G (OR = 1.5; 64.00 vs. 53.13%, *P* < 0.001, CI = 1.236–1.849), and SNP C159T (OR = 1.4; *P* = 0.001, 63.53 vs. 55.44%, CI = 1.148–1.708) (102). In a Turkish population the association between the CD14 -159C/T polymorphism and tuberculosis (88 patients vs. 116 control subjects) was investigated. There was no significant difference in terms of genotype distribution between patients with tuberculosis and controls, but serum levels of sCD14 were significantly increased in patients with active tuberculosis compared to those with inactive tuberculosis and healthy controls (*P* < 0.001) (104).

The most of associations of innate immune response genes and leprosy are summarized in Tables 1–4.

**Table 1:** **Summary of associations between genes of innate immune response *TLR*, *NOD2*, *PARK2*, *PACRG*, and leprosy**.

Genes	Population	SNP genotype/(risk allele)	Phenotype	Compared to	OR/(rr)	*P-*value	Conclusion
*TLR1*	Bangladeshi	N248S/SS/rs4833095	*Per se*	Controls	1.34	0.012	Susceptibility (14)
*TLR1*	Bangladeshi	N248S/SN/rs4833095	*Per se*	Controls	0.78	0.015	Protection (14)
*TLR1*	Bangladeshi	N248S/SS/rs4833095	ENL	Without ENL	0.21	–	Protection (14)
*TLR1*	Indian (New Delhi)	I602S/rs5743618	*Per se*	Controls	0.27	1.3 × 10^−6^	Protection (15)
*TLR1*	Indian (Kolkata)	I602S/SS/rs5743618	*Per se*	Controls	0,40	0.012	Protection (15)
*TLR1*	Turkish	I602S/SS/rs5743618	*Per se*	Controls	0.48	0.004	Protection (15)
*TLR1*	Brazilian	N248S/SS/rs4833095	*Per se*	Controls	1.81	0.004	Susceptibility (16)
*TLR2*	Ethiopian	N199N/rs3804099	RR	Controls	5.83	0.001	Susceptibility (19)
*TLR4*	Ethiopian	A299G/rs4986790-T390I/rs4986791	*Per se*	Controls	0.34/0.16	0.001	Protection (21)
*NOD2*	Chinese	rs9302752	Leprosy	Controls	2.28	1.42 × 10^−9^	Susceptibility (40)
*NOD2*	Chinese	rs7194886	Leprosy	Controls	2.25	4.43 × 10^−7^	Susceptibility (40)
*NOD2*	Vietnamese	rs9302752	Leprosy (family)	–	1.17	0.014	Susceptibility (43)
*NOD2*	Vietnamese	rs9302752	MB (family)	–	1.30	0.036	Susceptibility (43)
*NOD2*	Brazilian	rs8057341	Leprosy (family)	–	–	0.003	Protection (44)
*NOD2*	Brazilian	rs2111234	Leprosy (family)	–	–	0.031	Protection (44)
*NOD2*	Brazilian	rs3135499	Leprosy (family)	–	–	0.023	Protection (44)
*NOD2*	Brazilian	rs8057341-genotype AA	Leprosy	Controls	0.49	1.39 × 10^−6^	Protection (44)
*NOD2*	Brazilian	rs8057341-allele A	Leprosy and family	Controls	0.80	0.0001	Protection (44)
*NOD2*	Nepalese	rs12448797	Leprosy	Controls	3.20	0.016	Susceptibility (45)
*NOD2*	Nepalese	rs2287195	Leprosy	Controls	2.29	0.001	Susceptibility (45)
*NOD2*	Nepalese	rs8044354	Leprosy	Controls	2.17	0.001	Susceptibility (45)
*NOD2*	Nepalese	rs8043770	Leprosy	Controls	2.05	0.004	Susceptibility (45)
*NOD2*	Nepalese	rs13339578	Leprosy	Controls	2.19	0.001	Susceptibility (45)
*NOD2*	Nepalese	rs4785225	Leprosy	Controls	2.00	0.004	Susceptibility (45)
*NOD2*	Nepalese	rs751271	Leprosy	Controls	1.95	0.005	Susceptibility (45)
*NOD2*	Nepalese	rs1477176	Leprosy	Controls	–	0.0005	Susceptibility (45)
*NOD2*	Nepalese	rs8044354	ENL	(MB) without ENL	1.34	0.05	Susceptibility (45)
*NOD2*	Nepalese	rs17312836	ENL	(MB) without ENL	1.43	0.039	Susceptibility (45)
*NOD2*	Nepalese	rs1861759	ENL	(MB) without ENL	1.42	0.037	Susceptibility (45)
*NOD2*	Nepalese	rs1861758	ENL	(MB) without ENL	1.41	0.047	Susceptibility (45)
*NOD2*	Nepalese	rs1131716	LL	Controls	2.01	0.013	Susceptibility (45)
*NOD2*	Nepalese	rs2287195	RR	(PB) without RR	0.70	0.049	Protection (45)
*NOD2*	Nepalese	rs8043770	RR	(PB) without RR	0.68	0.028	Protection (45)
*NOD2*	Nepalese	rs7194886	RR	(PB) without RR	0.63	0.018	Protection (45)
*NOD2*	Nepalese	rs1861759	RR	(PB) without RR	0.66	0.027	Protection (45)
*PARK2*	Brazilian	PARK2_e01 (-2599) allele T	Leprosy	Controls	–	0.0003	Susceptibility (53)
*PARK2*	Vietnamese	PARK2_e01 (-2599) allele T	Leprosy	Controls	–	0.0006	Susceptibility (53)
*PARK2*	Brazilian	rs1040079 (allele C)	Leprosy	Controls	–	0.001	Susceptibility (53)
*PARK2*	Vietnamese	rs1040079 (allele C)	Leprosy	Controls	–	0.004	Susceptibility (53)
*PARK2/PARCRG*	Vietnamese	rs1333955 (C)	Leprosy	Controls	–	0.0007	Susceptibility (53)
*PARK2/PARCRG*	Brazilian	rs1333955 (C)	Leprosy	Controls	–	0.034	Susceptibility (53)
*PARK2/PARCRG*	Vietnamese	rs1333955 (C)	Leprosy (family)	–	–	0.004	Susceptibility (54)
*PARK2/PARCRG*	Indian	rs1333955 (C)	Leprosy	Controls	–	0.011	Susceptibility (54)
*PARK2/PARCRG*	Vietnamese	rs10806768 (A)	Leprosy (family)	–	–	0.012	Susceptibility (54)
*PARK2/PARCRG*	Indian	rs10806768 (A)	Leprosy	Controls	–	0.0438	Susceptibility (54)
*PARK2/PARCRG*	Vietnamese	rs6915128 (A)	Leprosy (family)	–	–	0.009	Susceptibility (54)
*PARK2/PARCRG*	Indian	rs6915128 (A)	Leprosy	Controls	–	0.0455	Susceptibility (54)
*PARCG*	Indian	rs10945859 (C)	Leprosy	Controls	1.32	0.0039	Susceptibility (57)
*PARK2*	Indian	rs9347683 (C)	Leprosy	Controls	1.31	0.0056	Susceptibility (57)
*PARK2*	Indian	rs9347684 (C)	Leprosy	Controls	1.29	0.0083	Susceptibility (57)
*PARK2*	Indian	rs9346929 (A)	Leprosy	Controls	1.31	0.0047	Susceptibility (57)
*PARK2*	Indian	rs4709648 (C)	Leprosy	Controls	1.23	0.055	Susceptibility (57)
*PARK2*	Indian	rs12215676 (C)	Leprosy	Controls	1.28	0.013	Susceptibility (57)
*PARK2*	Indian	rs10806765 (T)	Leprosy	Controls	1.32	0.0039	Susceptibility (57)
*PARK2*	Indian	rs6936373 (G)	Leprosy	Controls	1.26	0.023	Susceptibility (57)
*PARK2*	Indian	rs1333957 (A)	Leprosy	Controls	1.32	0.0034	Susceptibility (57)
*PARK2*	Indian	rs9365492 (C)	Leprosy	Controls	1.39	0.00036	Susceptibility (57)
*PARK2*	Indian	rs9355403 (A)	Leprosy	Controls	1.31	0.0047	Susceptibility (57)

*MB, multibacillary; PB, paucibacillary; BB, mid-borderline; BL, borderline lepromatous; BT, borderline tuberculoid; LL, lepromatous leprosy; TT, tuberculoid leprosy; *per se*, Leprosy independent of specific clinical manifestations; ENL, type 2 reactions or erythema nodosum leprosum; RR, Type I or reversal reaction; NR, leprosy who did not have reaction; UTR, untranslated region; OR, odds ratio; rr, relative risk; *P* values; SNPs, single-nucleotide polymorphisms; family, family-based association studies*.

**Table 2:** **Summary of associations between genes of innate immune response *MBL2*, *VDR*, *NRAMP1*, *MRC1*, and leprosy**.

Genes	Population	SNP/genotype/(allele)	Phenotype	Compared to	OR/(rr)	*P*-value	Conclusion
*MBL2*	Nepalese	G161A/rs1800450	LL	Controls	0.33	0.010	Protection (25)
*MBL2*	Brazilian	*LYPA*Y16577	Leprosy	Controls	2.25	0.003	Susceptibility (51)
*MBL2*	Brazilian	*LYPA*Y16577	LL	Controls	2.2	0.008	Susceptibility (51)
*MBL2*	Brazilian	*LYPA*Y16577	BB	Controls	2.98	0.008	Susceptibility (51)
*MBL2*	Chinese	(GC) rs11003125	MB	Controls	0.687	0.031	Protection (50)
*MBL2*	Chinese	(GC) rs7096206	PB	Controls	1.416	0.038	Susceptibility (50)
*MBL2*	Chinese	(AA) rs7100749	MB	Controls	9.091	0.044	Susceptibility (50)
*MBL2*	Chinese	(GA) rs7100749	PB	Controls	0.458	0.004	Protection (50)
*MBL2*	Chinese	(AC) rs11003124	Leprosy	Controls	1.357	0.038	Susceptibility (50)
*MBL2*	Chinese	(C) rs11003124	Leprosy	Controls	1.401	0.007	Susceptibility (50)
*MBL2*	Chinese	(C) rs11003124	MB	Controls	1.458	0.010	Susceptibility (50)
*VDR*	Brazilian	*Taq*I“tt”rs731236	Leprosy	MT (−)	13.33	–	Susceptibility (22)
*VDR*	Mexican	*Taq*I“TT”rs731236	LL	Controls	1.82	0.040	Susceptibility (26)
*VDR*	Indian	*Taq*I“tt”rs731236	TT	Controls	3.22	0.001	Susceptibility (23)
*VDR*	Indian	*Taq*I“TT”rs731236	LL	Controls	1.67	0.03	Susceptibility (23)
*VDR*	Indian	*Taq*I“Tt”rs731236	LL and TT	Controls	0.58	0.008	Protection (23)
*VDR*	Malawi	*Taq*I“tt”rs731236	Leprosy	Controls	4.3	0.004	Susceptibility (27)
*VDR*	Nepalese	Fok-I/rs2228570	RR	Without RR	1.31	0.032	Susceptibility (25)
*NRAMP1*	Brazilian	Genotype 23	Leprosy/MT (−)	HC/MT (−)	8.09	–	Susceptibility (29)
*NRAMP1*	Brazilian	Genotype 22 and 23	Leprosy/MT (−)	HC/MT (−)	7.03	–	Susceptibility (29)
*NRAMP1*	Vietnamese	–	Mitsuda	TDT	–	0.002	Susceptibility (30)
*NRAMP1*	Indonesian	*INT4*/469 + 14	PB	Controls	2.975	0.032	Susceptibility (31)
*NRAMP1*	Malian	3-UTR/1729 + 55del4	MB	PB	5.79	0.003	Susceptibility (32)
*NRAMP1*	Brazilian	274 C/T (TT)	ENH	Controls	–	0.04	Susceptibility (35)
*MRC1*	Vietnamese	G396S/rs1926736	*Per se*	Controls	0.76	0.035	Protection (37)
*MRC1*	Vietnamese	G396S/rs1926736	MB	Controls	0.71	0.034	Protection (37)
*MRC1*	Brazilian	G396Srs1926736	*Per se*	Controls	1.34	0.016	Susceptibility (37)
*MRC1*	Brazilian	G396Srs1926736	MB	Controls	1.42	0.023	Susceptibility (37)
*MRC1*	Brazilian	L407Frs2437257	*Per se*	Controls	0.75	0.09	Protection (37)
*MRC1*	Brazilian	L407F rs2437257	MB	Controls	0.63	0.04	Protection (37)
*MRC1*	Chinese	(CT) rs692527	PB	Controls	0.598	0.022	Susceptibility (38)
*MRC1*	Chinese	(TT) rs34856358	PB	Controls	1.688	0.022	Susceptibility (38)

*MB, multibacillary; PB, paucibacillary; BB, mid-borderline; BL, borderline lepromatous; BT, borderline tuberculoid; LL, lepromatous leprosy; TT, tuberculoid leprosy; *per se*, leprosy independent of specific clinical manifestations; ENL, type 2 reactions or erythema nodosum leprosum; RR, type I or reversal reaction; NR, leprosy who did not have reaction; UTR, untranslated region; OR, odds ratio; rr, relative risk; *P* values; SNPs, single-nucleotide polymorphisms; HC, healthy contacts; MT (−), Negative Mitsuda test*.

**Table 3:** **Summary of associations between genes of innate immune response *MICA*, *MICB*, *KIR*, *TNF*, *LTA*, *BAT1*, *IFNG*, and leprosy**.

Genes	Population	SNP allelic group/genotype ID	Phenotype	Compared to	OR/(rr)	*P-*value	Conclusion
*MICA*	Chinese	MICA*A5	MB	Controls	(0.52)	<0.05	Protection (60)
*MICA*	Indian	MICA*5A5.1	Leprosy (family)	–	–	0.006	Susceptibility (58)
*MICA/MICB*	Indian	MICB*CA16	Leprosy (family)	–	–	0.031	Susceptibility (58)
*MICA/MICB*	Indian	MICB*CA19	Leprosy (family)	–	–	0.021	Susceptibility (58)
*MICA/MICB*	Indian	MICB*CA21	Leprosy (family)	–	–	0.015	Protection (58)
*MICA*	Brazilian	MICA*027	Leprosy	Controls	0.37	0.02	Protection (62)
*MICA*	Brazilian	MICA*027	MB	Controls	0.27	0.01	Protection (62)
*MICA*	Brazilian	MICA*010	MB	Controls	0.35	0.05	Protection (62)
*KIR*	Brazilian	KIR2DS3	TT	LL	2.72	0.0422	Susceptibility (65)
*KIR*	Brazilian	KIR2DL1-C2	BB	Controls	0.48	0.0350	Protection (65)
*KIR*	Brazilian	KIR2DL1-C2	BB	TT	0.30	0.0197	Protection (65)
*KIR*	Brazilian	KIR2DL3-C1	TT	Controls	0.43	0.0231	Protection (65)
*KIR*	Brazilian	KIR3DL2-A3/11	BB	Controls	2.04	0.048	Susceptibility (65)
*KIR*	Brazilian	KIR2DL1	Leprosy	Controls	0.3	0.014	Protection (66)
*KIR*	Brazilian	KIR2DL1	BB	Controls	0.2	0.014	Protection (66)
*KIR*	Brazilian	KIR2DS2-C1	Leprosy	Controls	1.4	0.031	Susceptibility (66)
*KIR*	Brazilian	KIR2DS2-C1	TT	Controls	1.9	0.045	Susceptibility (66)
*KIR*	Brazilian	KIR2DL2-C1	Leprosy	Controls	2.6	0.024	Susceptibility (66)
*KIR*	Brazilian	KIR2DL2-C1	TT	Controls	4.1	0.045	Susceptibility (66)
*KIR*	Brazilian	KIR2DL2-C1	LL	Controls	7.5	0.032	Susceptibility (66)
*TNF*	Thailand	TNF-G-308 (A)	Leprosy	Controls	0.52	0.016	Protection (25)
*TNF*	Thailand	TNF-308 (G/A)	Leprosy	Controls	2.69	0.04	Susceptibility (33)
*TNF*	Thailand	TNF-308 (A)	MB	Controls	2.93	0.04	Susceptibility (33)
*TNF*	Brazilian	TNF-308	*Per se* (family)	–	–	0.000001	Susceptibility (68)
*TNF/LTA*	Brazilian	TNF*1/LTA*2	*Per se* (family)	–	–	0.014	Susceptibility (68)
*TNF/LTA*	Brazilian	TNF*2/LTA*2	*Per se* (family)	–	–	0.001	Protection (68)
*BAT1*	Indian	rs2523504	Leprosy	Controls	1.48	2.5 × 10^6^	Susceptibility (77)
*LTA*	Indian	rs13192469	Leprosy	Controls	1.58	7.2 × 10^7^	Susceptibility (77)
*TNF*	Indian	rs1800610	Leprosy	Controls	1.45	2.8 × 10^4^	Susceptibility (77)
*IFNG*	Brazilian	IFNG + 874(T) rs2430561	Leprosy	Controls	0.75	0.005	Protection (79)
*IFNG*	Chinese	(10CA) rs3138557	Leprosy	Controls	4.202	0.001	Susceptibility (38)
*IFNG*	Chinese	(13CA) rs3138557	MB	Controls	1.435	0.026	Susceptibility (38)
*IFNG*	Chinese	(15CA) rs3138557	MB	Controls	1.369	0.007	Susceptibility (38)
*IFNG*	Chinese	(17CA) rs3138557	PB	Controls	2.239	0.040	Susceptibility (38)
*IFNG*	Brazilian	IFNG + 874(AA)	PB	MB	1.62	0.028	Susceptibility (80)
*IFNG*	Brazilian	alleles 5, 6, and 7	TT	Controls	–	0.013	Susceptibility (81)

*MB, multibacillary; PB, paucibacillary; BB, mid-borderline; BL, borderline lepromatous; BT, borderline tuberculoid; LL, lepromatous leprosy; TT, tuberculoid leprosy; *per se*, Leprosy independent of specific clinical manifestations; ENL, type 2 reactions or erythema nodosum leprosum; RR, type I or reversal reaction; NR, leprosy who did not have reaction; UTR, untranslated region; OR, odds ratio; rr, relative risk; *P* values; SNPs, single-nucleotide polymorphisms; Family, family-based association studies*.

**Table 4:** **Summary of associations between genes of interleukins and leprosy**.

Genes	Population	SNP allelic group/genotype ID/haplotype	Phenotype	Compared to	OR/(rr)	*P-*value	Conclusion
*IL-6*	Brazilian	(CC) rs1800795	ENL	NR	2.41	0.03	Susceptibility (83)
*IL-6*	Brazilian	(CC + CG) rs1800795	ENL	NR	3.71	0.005	Susceptibility (83)
*IL-6*	Brazilian	(AA) rs2069832	ENL	NR	2.71	0.007	Susceptibility (83)
*IL-6*	Brazilian	(AA + AG) rs2069832	ENL	NR	4.00	0.002	Susceptibility (83)
*IL-6*	Brazilian	(GG) rs2069840	ENL	NR	0.44	0.03	Protection (83)
*IL-6*	Brazilian	(GG + CG) rs2069840	ENL	NR	0.39	0.04	Protection (83)
*IL-6*	Brazilian	(GG) rs2069845	ENL	NR	1.92	0.04	Susceptibility (83)
*IL-6*	Brazilian	(GG + AG) rs2069845	ENL	NR	2.59	0.045	Susceptibility (83)
*IL-12B*	Indian	3′UTR2.2	Leprosy	Controls	–	0.001	Protection (85)
*IL-12*	Mexican	3′UTR 1188 A/C (CC)	LL	Controls	–	<0.05	Susceptibility (86)
*IL-12*	Mexican	3′UTR 1188 A/C (AC)	LL	Controls	–	<0.05	Protection (86)
*IL12B*	Chinese	rs6871626	Leprosy	Controls	0.75	3.95 × 10^18^	Protection (88)
*IL18RAP*	Chinese	rs2058660	Leprosy	Controls	1.30	4.57 × 10^19^	Susceptibility (88)
*IL12B*	Indian	(AA vs. AC + CC) rs2853694	Leprosy	Controls	1.42	2.6 × 10^4^	Susceptibility (89)
*IL-10*	Brazilian	819T	Leprosy	Controls	1.35	0.03	Susceptibility (91)
*IL-10*	Brazilian	819TT	Leprosy	Controls	2.64	0.04	Susceptibility (69)
*IL-10*	Brazilian	819T	PB	MB	2.28	0.01	Susceptibility (69)
*IL-10*	Colombian	(C/C and C/T) rs1800871	Leprosy	Controls	4.34	<0.001	Susceptibility (92)
*IL-10*	Colombian	(C/C and C/A) rs1800872	Leprosy	Controls	4.3	<0.001	Susceptibility (92)
*IL-10*	Colombian	Haplotype (819C-519C)	Leprosy	Controls	4.34	<0.001	Susceptibility (92)
*IL-10*	Colombian	1082A-819C-592C	Leprosy	Controls	6.25	<0.001	Susceptibility (92)
*IL-10*	Brazilian	-3575-2849-2763	Leprosy	Controls	–	0.044	Susceptibility (93)
*IL-10*	Brazilian	-3575A-2849G-2763C	Leprosy	Controls	0.35	0.005	Protection (93)
*IL-10*	Indian	-819 (TT vs. CT + CC)	Leprosy	Controls	2.50	<0.005	Susceptibility (94)
*IL-10*	Indian	-819 (CC vs. CT + TT)	Leprosy	Controls	0.59	0.005	Protection (94)
*IL-10*	Indian	-592 (AA vs. CA + CC)	Leprosy	Controls	2.43	<0.005	Susceptibility (94)
*IL-10*	Indian	-592 (CC vs. CA + AA)	Leprosy	Controls	0.60	0.006	Protection (94)
*IL-10*	Indian	-3575T-2849G-2763C-1082A-819C-592C	Leprosy	Controls	0.58	0.01	Protection (94)
*IL-10*	Brazilian	1082G-819C-592C	LL	Controls	–	0.02	Protection (70)

*MB, multibacillary; PB, paucibacillary; BB, mid-borderline; BL, borderline lepromatous; BB, borderline tuberculoid; LL, lepromatous leprosy; TT, tuberculoid leprosy; *per se*, leprosy independent of specific clinical manifestations; ENL, type 2 reactions or erythema nodosum leprosum; RR, type I or reversal reaction; NR, leprosy who did not have reaction; UTR, untranslated region; OR, odds ratio; rr, relative risk; *P* values; SNPs, single-nucleotide polymorphisms*.

## Conclusion

The studies discussed in this review were conducted in diverse populations where the incidence of the disease is still high. Although the results are consistent with the biological functions of their polymorphic genes, many studies present a number of samples down to be regarded as true and consistent science. This would need from the data shown in the literature, a continuation of research aimed at understanding the mechanisms of infection and defense to *M. leprae* with larger number of samples and selected controls (household contacts), and later of great relevance, replication of the main findings in other populations the incidence of the disease remains high, giving continuation to the transmission chain.

It will be a great advance to define in the near future, the exact polymorphisms that lead to the clinical outcome of leprosy, and that make individuals possessing up of resistant form to infection or even spontaneous healing. Most findings are also presented in a simplified manner without further deepening the understanding of the disease outcome, only indicating a possible influence in leprosy, so parking not completing a broader range with concrete explanations and proven based on studies standards case–control, statistical rules, the local incidence of the disease investigated and can sometimes generate false positives, slowing the scientific understanding of the disease.

## Future Perspectives

Whereas the intervention model for disease control is based on early diagnosis, timely treatment of all diagnosed cases, prevention and treatment of disabilities and surveillance of household contacts; genetic characterization of gene polymorphisms of the immune response of a population that developed leprosy, will investigate the influence of these genes in the resistance or susceptibility to disease and\or clinical forms, and can generate a characteristic genetic profile of patients and controls in this way we can redirect drug therapy according to the individual’s profile. This step may be essential to prevent the development of leprosy reactions, because the patient be diagnosed as early as possible and with the most appropriate medication their clinical form. Another advantage of the characterization of genetic patient profile will be compared to their close contacts, so we can see very clearly where there was immunological failure, and the profiles are similar, you can investigate the replacement of the drugs used in order to avoid the development of leprosy in individuals with susceptible profiles. Thus, studies are needed with genes and their receptors from the recognition of the microorganism to the outcome of the disease to determine the involvement of these genes in the immunopathogenesis of leprosy patients and not consanguineous individuals who live together next.

## Conflict of Interest Statement

The author declares that the research was conducted in the absence of any commercial or financial relationships that could be construed as a potential conflict of interest.
